# Fitness effects for *Ace* insecticide resistance mutations are determined by ambient temperature

**DOI:** 10.1186/s12915-020-00882-5

**Published:** 2020-10-30

**Authors:** Anna Maria Langmüller, Viola Nolte, Ruwansha Galagedara, Rodolphe Poupardin, Marlies Dolezal, Christian Schlötterer

**Affiliations:** 1 Institut für Populationsgenetik, Vetmeduni Vienna, Veterinärplatz 1, 1210 Vienna, Austria; 2 Vienna Graduate School of Population Genetics, Vetmeduni Vienna, Veterinärplatz 1, 1210 Vienna, Austria; 3 Present Address: Paracelsus Medical University Salzburg, Strubergasse 21, 5020 Salzburg, Austria; 4 Plattform Bioinformatik und Biostatistik, Vetmeduni Vienna, Veterinärplatz 1, 1210 Vienna, Austria

**Keywords:** Drosophila, Experimental Evolution, Insecticide Resistance, Temperature

## Abstract

**Background:**

Insect pest control programs often use periods of insecticide treatment with intermittent breaks, to prevent fixing of mutations conferring insecticide resistance. Such mutations are typically costly in an insecticide-free environment, and their frequency is determined by the balance between insecticide treatment and cost of resistance. *Ace*, a key gene in neuronal signaling, is a prominent target of many insecticides and across several species, three amino acid replacements (I161V, G265A, and F330Y) provide resistance against several insecticides. Because temperature disturbs neuronal signaling homeostasis, we reasoned that the cost of insecticide resistance could be modulated by ambient temperature.

**Results:**

Experimental evolution of a natural *Drosophila simulans* population at hot and cold temperature regimes uncovered a surprisingly strong effect of ambient temperature. In the cold temperature regime, the resistance mutations were strongly counter selected (*s* = − 0.055), but in a hot environment, the fitness costs of resistance mutations were reduced by almost 50% (*s* = − 0.031). We attribute this unexpected observation to the advantage of the reduced enzymatic activity of resistance mutations in hot environments.

**Conclusion:**

We show that fitness costs of insecticide resistance genes are temperature-dependent and suggest that the duration of insecticide-free periods need to be adjusted for different climatic regions to reflect these costs. We suggest that such environment-dependent fitness effects may be more common than previously assumed and pose a major challenge for modeling climate change.

## Background

Acetylcholinesterase (AChE) is a well-studied insecticide target that is involved in the breakdown of the neurotransmitter acetylcholine. It is targeted by organophosphates and carbamates [1], which are widely used all over the world. Soon after their introduction in the 1950s and 1960s, the first cases of insecticide resistance alleles of the *Ace* gene were reported [2]. In many arthropod species, insecticide resistance is mediated by insensitive *Ace* alleles [3–6]. A haplotype containing three resistance mutations (I161V, G265A, F330Y) occurs worldwide at high frequencies in *Drosophila melanogaster* [7] and provides higher levels of resistance to insecticides than haplotypes that contain only one or two out of the three resistance mutations. The same three resistance mutations were also identified in *Drosophila simulans* [8]. Such new resistance mutations are generally assumed to be deleterious in the absence of insecticides [9]. Considerable fitness costs have been inferred for *Ace* resistance alleles [10, 11], which can arise from various factors, such as reduced substrate affinity, reduced chemical turnover, or lower protein stability [10].

**Fig. 1:**
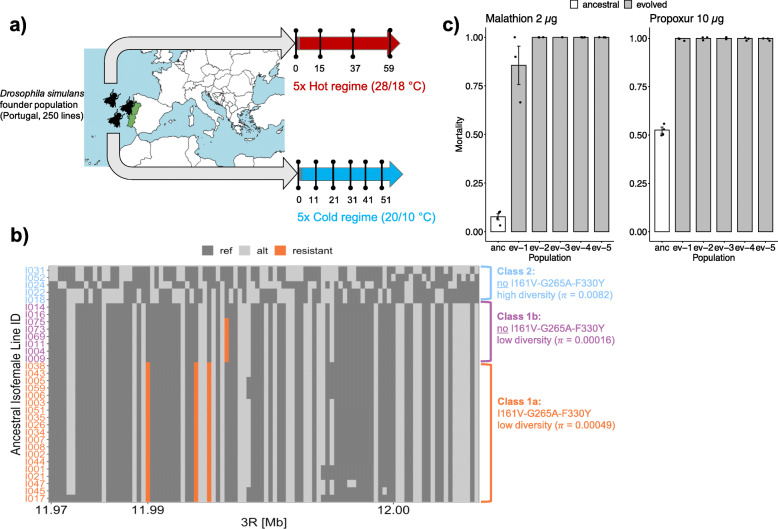
Experimental design and characteristics of the founder population. **a** The ancestral experimental population was composed of 250 isofemale lines from a natural Portuguese *D. simulans* population. Five replicated experimental populations were maintained at two temperature regimes. In the hot regime (red), populations are exposed to 12 h of light (28 °C) and 12 h of darkness (18 °C). In the cold regime (blue), populations are exposed to 12 h of light (20 °C) and 12 h of darkness (10 °C). All populations were maintained at a constant census size (at least 1000 flies) and non-overlapping generations. Genomic sequences (Pool-seq [13]) were obtained from the ancestral population (generation 0) and at generations 15, 37, and 59 for the hot regime and generations 11, 21, 31, 41, and 51 for the cold regime. **b** Haplotype structure at the *Ace* locus: The *x*-axis shows the genomic position on chromosome 3R in Mb around the *Ace* locus. Each single row shows the haplotype information of one isofemale line originating from the natural Portuguese *D. simulans* population. Isofemale line identifiers are colored by the assigned haplotype class (2 in blue, 1a in orange, and 1b in magenta). Each column represents one exonic SNP at the *Ace* locus. Reference alleles (M252) are colored in dark gray and alternative alleles in light gray. The four previously reported resistant mutations I161V, G265A, F330Y, and G368A [7] are highlighted in orange, with I161V/G265A/F330Y being specific to haplotype class 1a. **c** Single-dose bioassays for malathion (left column) and propoxur (right column) for the reconstituted ancestral population (anc) and five evolved replicates from the hot regime (ev-1 to ev-5, generation 132). Bars show the average mortality (24 h after insecticide exposure) for each experimental population; the error bars show the standard error of the mean

Understanding the fitness consequences of insecticide resistance mutations is of key interest for management strategies. With temperature being a major challenge for neuronal signaling homeostasis [12] and AChE serving a central role in neuronal signaling, we reasoned that the fitness consequences of the *Ace* insecticide resistance alleles may be modulated by ambient temperature. We tested this hypothesis by experimental evolution in an insecticide-free environment. We exposed a natural *D. simulans* population to either a cold environment fluctuating between 10 and 20 °C or a hot environment fluctuating between 18 and 28 °C (Fig. 1a) and followed the allele frequency changes of *Ace* resistance alleles for 51 generations in five replicates in the cold regime and 59 generations in five replicates in the hot regime. This provided the opportunity to determine the evolutionary response under a stable temperature regime over much longer times than typically possible in natural populations.

## Results

The *D. simulans* founder population, which is a sample from a natural Portuguese population, has a pronounced haplotype structure at the *Ace* locus (Fig. 1b). Haplotypes belonging to the most abundant haplotype class (class 1) were highly similar with very few segregating variants (*π* = 0.0004). The second haplotype class (class 2) showed normal levels of variation (*π* = 0.0082) [14] and no apparent pattern of linkage disequilibrium (Fig. 1b). The highly reduced variability of the first haplotype class suggests recent strong selection, as expected for insecticide resistance alleles. The majority (59%) of the sequences carried the same three insecticide resistance mutations (I161V, G265A, F330Y) that are also highly abundant in *D. melanogaster* [7]. No haplotype with only a subset of these variants was detected. We further identified an additional resistance mutation (G368A), which was also described for *D. melanogaster* [7]. This resistance allele was not detected on haplotypes carrying the three other resistance mutations (Fig. 1b) and segregated at a frequency of 31% in the founder population (frequency estimates are based on Pool-seq data).

The founder population was highly resistant against two different classes of insecticides, carbamate (propoxur) and organophosphate (malathion), both targeting AChE [1]. Consistent with a significant cost of insecticide resistance mutations, insecticide resistance was strongly reduced in all replicates after evolution in the insecticide-free laboratory environment (Fig. 1c). Resistance levels of the evolved populations were similar to the strain M252 from Madagascar, the presumed origin of *D. simulans*, which does not carry any resistance mutation at the *Ace* locus (M252 LD_50_ propoxur = 0.5 μg, M252 LD_50_ malathion = 0.1 μg) [15]. We used Pool-seq [13] to follow the allele frequency trajectories in all replicates of the two temperature regimes. On the genomic level, we find a pronounced frequency drop of the three resistance mutations (I161V, G265A, F330Y) at generation 51 in the cold regime and generation 59 in the hot regime (on average 28.79%). The heterogeneous trajectories of the resistance variant G368A across replicates does, however, not result in a mean frequency change (2% in the hot regime, 1% in the cold regime). We conclude that I161V, G265A, and F330Y come with a cost in insecticide-free environments, but the mutation G368A does not. Furthermore, the resistance assays do not show a significant effect of G368A because evolved populations were highly susceptible to the tested insecticides, similar to M252, which lacks this mutation. In contrast to the I161V/G265A/F330Y triple mutant, the single G368A mutation was reported previously to provide resistance to only a moderate number of insecticides (e.g., not against propoxur) [7], which could explain why evolved populations are highly susceptible although G368A is segregating. We did not consider the mutation G368A in further analyses.

**Fig. 2:**
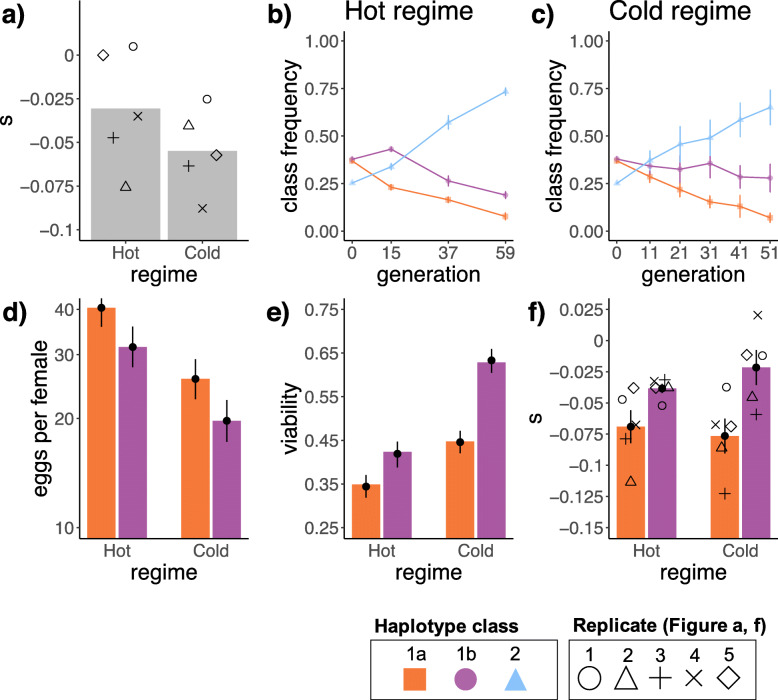
Genomic and phenotypic analysis. **a** Selection coefficient of the three resistance mutations (I161V, G265A, and F330Y) in the two temperature regimes determined by the comparison of haplotype class 1a to class 1b. The selection coefficient was defined as *s*_1*a*_ − *s*_1*b*_. Bars show the average selection coefficient of the three resistance mutations over five replicated experimental populations; single points show the selection coefficient for individual experimental replicates. **b** Haplotype class trajectories in the hot regime. In each experimental population, the haplotype class frequency is determined by the median frequency of all marker SNPs. Dots show the haplotype class frequency averaged across replicated experimental populations; error bars indicate the standard error of the mean (see Additional file 1: Figure S1 for replicate-specific haplotype class trajectories). **c** Haplotype class trajectories in the cold regime (see Additional file 1: Figure S2 for replicate-specific haplotype class trajectories). **d** Average fecundity in the two temperature regimes, for haplotype classes 1a and 1b. The fitted model (log_10_ transformed eggs per female) is shown as black dots with 95% confidence intervals (determined by parametric bootstrapping; *n* = 1000). **e** Average viability estimated as the proportion of eclosed flies in the two temperature regimes, for haplotype classes 1a and 1b. The fitted model is shown as black dots with 95% confidence intervals (parametric bootstrapping; *n* = 1000). **f** Selection coefficients of the two haplotype classes differing only by the presence/absence of the three resistance mutations (1a, 1b) in the two temperature regimes. Selection coefficients were estimated for the haplotype class frequencies (= median frequency of all marker SNPs) in each of the five replicated experimental populations. Colored bars show the average selection coefficients, error bars show the standard error of the mean among replicated experimental populations, and single points show the selection coefficients for individual experimental replicates

The special haplotype structure in the founder population provided an excellent framework to further test the fitness effects of insecticide resistance. The two highly similar haplotype classes (1a, 1b) which are segregating in the founder population differ by the presence/absence of the three resistance mutations. The relative frequency change of the two haplotype classes therefore provides a direct readout of their relative fitness. Because the same founder population evolved in hot and cold environments, it is possible to determine the temperature dependence of the fitness costs. In the cold temperature regime, we observed the expected fitness cost of the resistance alleles (*s* = − 0.055). However, in the hot temperature regime, the fitness costs of the three resistance mutations decreased by almost 50% (*s* = − 0.031) (Fig. 2a), indicating that temperature modulates the effect of the resistance mutations in an insecticide-free environment.

The temperature-dependent fitness effect of the three resistance mutations was inferred in the presence of a third haplotype class (class 2), which is the fittest haplotype class, independent of environmental temperature (Fig. 2b, c; Additional file 1: Figure S1, and S2). To rule out that an interaction with haplotype class 2 is responsible for this discovery, we confirmed the relative fitness change of haplotype class 1a and 1b in an independent experiment. We measured the fitness components fecundity and egg to adult viability for eleven isofemale lines from the founder population that were homozygous at the *Ace* locus but differed in the presence/absence of the resistance mutations. Because we observed in the founder population only haplotypes with either all three resistance mutations or none, we focused on these two haplotype classes. We expect a significant interaction between haplotype class and temperature regime for at least one fitness component, if the fitness cost of the three resistance mutations is temperature-dependent. Unlike fecundity (Fig. 2d, Additional file 1: Table S1), we found a significant interaction between haplotype class and temperature regime for the viability fitness component (full-null model comparison *χ*^2^ = 14.479, df = 1, *p* < 0.001, Additional file 1: Table S2) with haplotype class 1b performing better in the cold regime (Fig. 2e). The high consistency between viability and the selection strength inferred by experimental evolution (Fig. 2a, f) confirms that the cost of insecticide resistance is temperature-dependent.

Another very interesting pattern emerged from the highly polymorphic haplotype class 2. While having the lowest frequency in the founder population, this haplotype class dominated the evolved populations, independent of the temperature regime (Fig. 2b, c; Additional file 1: Figure S1, and S2). The selective advantage of this haplotype class was on average 1.7 times higher than the absolute fitness difference between haplotype classes with and without insecticide resistance. Thus, the two haplotype classes without the three resistance mutations had very different fitness. Why haplotype class 2 outperformed all other haplotype classes in the experimental evolution setting, but not in the wild, requires further investigations.

## Discussion

### Temperature modulates fitness effects of *Ace* insecticide resistance mutations

We show that haplotype class 1a and haplotype class 1b, which mainly differ by the presence of three insecticide resistance mutations, have different fitness costs at hot and cold temperatures (Fig. 2a, f). Such temperature-specific fitness effects have been noted before for example in mosquitos [16], the Australian sheep blowfly *Lucilia cuprina* [17], the aphid *Myzus persicae* [18], and the grasshopper *Melanoplus differentialis* [19]*.* A major challenge for the unambiguous demonstration of temperature-specific effects in natural populations is confounding factors, such as environmental heterogeneity and migration [16, 20]. Experimental evolution studies overcome these limitations, but their relevance has been challenged due to a benign environment [21, 22]. The temperature stress used in this study is one strategy to reduce this potential caveat. Our results are a vivid demonstration that it is not sufficient to focus on the molecular mutations, but environmental factors, such as temperature, need to be included to understand fitness costs [21, 23–29].

### Insecticide resistance as a driver of temperature adaptation

**Fig. 3:**
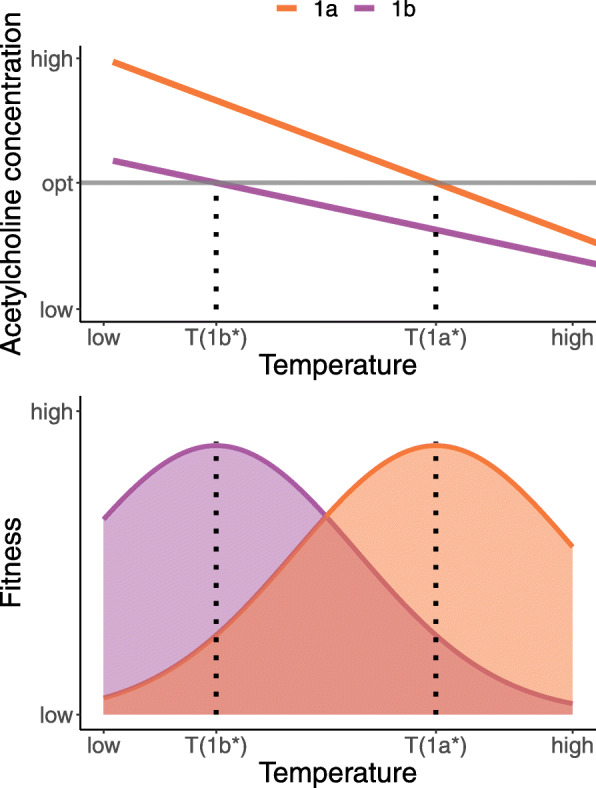
Schematic overview of resistance mutations with temperature-specific effects. An increased activity of AChE at higher temperature results in a major challenge for neuronal signaling homeostasis because acetylcholine is degraded at higher levels (top panel). Because insecticide resistance mutations destabilize AChE, less acetylcholine is degraded in individuals carrying the resistance mutations (1a), independent of the temperature. In the hot, this provides them with a fitness advantage compared to individuals without the destabilizing resistance mutations (1b) since those suffer from the excess activity of the fully functional enzyme at high temperature. The inverse applies to the cold environment in which the fully functional 1b haplotype class can better compensate for the reduced enzymatic activity. Because the two alleles result in AChE activity which is closer to the optimum at different temperatures, the fitness of the two alleles differs at hot and cold temperatures (bottom panel)

The pronounced reduction in fitness cost of the three resistant mutations in the hot relative to the cold environment (Fig. 2a) is particularly interesting. We reason that this temperature-specific behavior is related to enzyme activity. A higher activity of AChE in the hot provides a major challenge for signaling homeostasis. Thus, alleles that reduce the excess activity of AChE in hot environments may be less deleterious at this temperature regime. While the I161V/G265A/F330Y triple mutant has been reported to have a similar enzymatic activity to the wild type, the reduced protein stability caused by the three resistance mutations will decrease the amount of functional AChE in the synaptic cleft [10] and therefore counter the effects of hot temperature and facilitate signaling homeostasis. On the other hand, the already lower enzymatic activity in the cold explains why the resistance mutations which further reduce the efficiency of AChE are highly deleterious in the cold (Fig. 3).

It is remarkable that a different component of neuronal signaling, the dopamine pathway, has been identified as a major driver of adaptation in an experimental evolution study using the same temperature regime, but a different founder population [30]. We propose that neuronal signaling is a major target of temperature adaptation, and the evolutionary response depends on the selection targets which require specific changes to retain signaling homeostasis [23]. Our hypothesis is further supported by clinal variation in natural *D. simulans* populations for these three resistance mutations in Australia, with higher frequencies in hot environments [8]. In *D. melanogaster*, *Ace* shows also clinal variation [31] and is among the most differentiated genes in natural populations [32]. Nevertheless, variation in insecticide treatment could also explain this pattern and very likely operates synergistically with the discovered fitness advantage in hot environments.

### In vitro experiments cannot be generalized to estimate in vivo fitness costs

In vitro methods suggested that most single mutations impose a cost of reduced activity and stability, but the combination of three mutations provides a higher resistance at a minimized fitness costs which suggests that the triple mutant I161V/G265A/F330Y may segregate in natural populations in the absence of insecticides [10]. Contrary to these in vitro predictions, we find that the three linked resistance mutations suffer from severe fitness costs in the cold temperature regime, while the single mutation G368A did not decrease in frequency across both temperature regimes. This finding highlights that in vivo fitness measurements in evolving populations are crucial to complement and test predictions based on in vitro experiments, which are often performed at one constant temperature regime [10]. Further experiments are needed to test if the observed differences reflect the full organismal complexity or whether in vitro experiments need to be performed under a broader range of conditions.

### Fitness inference in complex populations is difficult

**Fig. 4:**
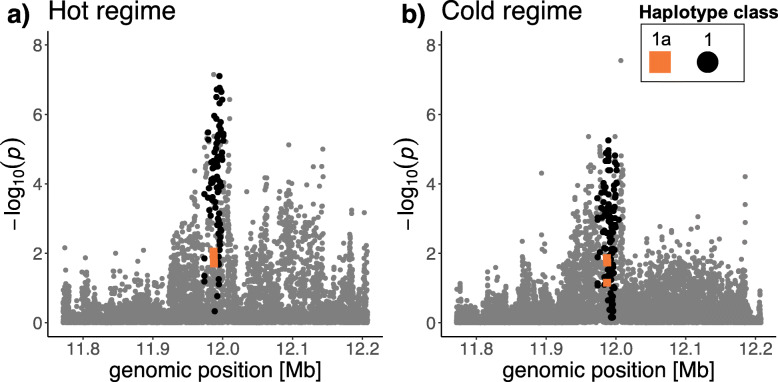
Manhattan plots around the *Ace* locus. **a** −log_10_ transformed *p* values of a CMH test for Pool-seq data (*y*-axis) between generations 0 and 59 (hot regime) around the *Ace* locus. The three resistance mutations I161V, G265A, and F330Y are highlighted in orange and marker SNPs of class 1 (= 1a + 1b) in black. A genomic region of 200 kb up- and downstream of *Ace* is shown. **b** −log_10_ transformed *p* values of a CMH test for Pool-seq data (*y*-axis) between generations 0 and 51 (cold regime) around the *Ace* locus

The combination of a priori known selection targets with time series data and haplotype information provides an extremely powerful setting to uncover the complexities of selection. Independent of the temperature-specific fitness effects of the three resistance mutations, we find considerable heterogeneity in fitness among the haplotype classes lacking the three resistance mutations—haplotype class 1b is outcompeted by haplotype class 2 independent of the temperature regime. Such haplotype-specific fitness is not unique to our experiment but has been noted before for susceptible haplotypes [33]. Because the haplotype classes share segregating variants, their heterogeneous fitness complicates the interpretation of selection signatures, which is nicely illustrated by the analysis of all SNPs in *Ace*. Despite the significant cost imposed by the three resistance mutations, they are not the top candidate SNPs for the selection target, even in the cold environment. The reason is that haplotype class 2 is fitter than both haplotype class 1a and class 1b. As a consequence, the analysis of individual SNPs detects a stronger selection signature for variants shared between 1a and 1b than for the resistance mutations, which are restricted to haplotype class 1a (Fig. 4). Only when the three haplotype classes are considered separately, the complexity of the selection history can be detected. Hence, we propose that selection is more reliably inferred in complex, polymorphic populations on the haplotype level than using independent SNPs.

## Conclusions

Precise estimates of fitness costs and a profound understanding of how these fitness costs are influenced by environmental factors such as temperature are key to a successful insecticide management strategy [28]. Our study confirmed that temperature is a key factor determining the costs of resistance. The failure to predict the fitness cost based on in vitro experiments nicely demonstrates the need to study resistance costs in evolving populations. More work is needed to determine whether well-designed experimental evolution experiments can predict the dynamics of resistance mutations in the wild.

## Methods

### Experimental *D. simulans* populations

A detailed description of the experimental setup can be found in Mallard et al. [34]. In brief, ten replicated populations were created from 250 isofemale lines that originated from a wild *D. simulans* population in Portugal (sampled in 2008). Five replicates each were randomly assigned to one of two different thermal selection regimes: a hot regime (12-h light and 28 °C; 12-h dark and 18 °C) and a cold regime (12-h light and 20 °C; 12-h dark and 10 °C). Apart from different ambient temperatures, the populations were maintained in the same way with non-overlapping generations and a census size of 1000 individuals.

### Pool-seq analysis

The details of the genomic sequencing can be found in Mallard et al. [34] and in Otte et al. [35]. Briefly, the founder population for the Evolve and Resequence experiment was sequenced with the Pool-seq approach [13], as were generations 15, 37, and 59 for the hot regime and generations 11, 21, 31, 41, and 51 for the cold regime. Raw paired-end reads were trimmed [36] and mapped against the *D. simulans* reference genome [15] with three different mapping algorithms (novoalign [37], bwa-mem [38], and bowtie2 [39]) to assure a robust SNP set [40]. Mapped reads were filtered for a mapping quality of at least 20 and proper pairs using SAMtools [41], and duplicates were removed from the data [42]. Filtered bam files were transformed to mpileup [41] and sync format [43]. The resulting sync files were masked for indels [43], transposable elements, repeats [44, 45], and known Y-translocations [46] using Popoolation2 [43]. SNPs were filtered for a minor allele count of at least 5, a mapping quality of at least 30, and had to have consistent allele frequency estimates over the three different mapping algorithms. A modified Cochran-Mantel-Haenszel test (CMH-test) which accounts for genetic drift and Pool-Seq noise [47] was used to identify selected SNPs. The CMH-test allows to test for independence of matched data—i.e., allele counts for ancestral and evolved populations [48]. Effective population sizes of the experimental populations were calculated with the R package poolSeq [49]. These effective population size estimates were used in the haplotype class analysis mentioned below.

### Experimental haplotype inference

Since Pool-seq provides linkage information only up to the read length [13], genotyped individuals are necessary to assess the linkage structure at the *Ace* locus. We experimentally derived 32 haplotypes from the ancestral population as described previously [50] by crossing one individual male from an isofemale line with a virgin female from the inbred reference strain [15]. For each cross, we used a single female offspring to extract genomic DNA with a high-salt protocol [51]. Between 50 and 120 ng genomic DNA were fragmented with a Covaris S2 Focused-ultrasonicator (Covaris, Inc. Woburn, MA, USA), and Illumina sequencing libraries were prepared using either the TruSeq v2 DNA Sample Prep Kit (Illumina, San Diego, CA) or the NEBNext Mastermix Kit (E6040L) (New England Biolabs, Ipswich, MA) with single-index adapters. Library fragments with an approximate insert size of 330 bp were selected using either agarose gel or AMPureXP beads (Beckman Coulter, Carlsbad, CA), and barcoded libraries were amplified with 10 PCR cycles. After combining them into pools with 12 samples each, 2 × 100 bp paired-end reads were sequenced on two or three lanes for each pool on a HiSeq 2000.

Raw paired-end reads were trimmed with a java implementation of trim-fastq.pl (--quality-threshold 18, --min-length 50, --no-5p-trim) [52] and mapped to the *D. simulans* reference genome [15] with bwa aln [38] (v.0.7.12-r1039, -o 1 –n 0.01 –l 200 –e 12 –d -1) [52]. Barcoded files were split with an in-house java script allowing 1 mismatch. Reads that mapped 200 kb up- and downstream of the *Ace* locus were extracted (bp 11 771 451 – 12 207 715, samtools (v.1.9)) [41]. Mapped reads were filtered for duplicates [42], a mapping quality of at least 20, proper pairs (samtools, v.1.9) [41], and overlapping mates from read pairs were clipped with BamUtil clipOverlap (v.1.0.13) [53].

Polymorphisms were called with freebayes (--use-best-n-alleles 4, v. 1.3.1) [54], masked for repeats, known Y translocations [44, 46], and polymorphisms in 5 bp proximity to indels (bcftools v.1.9) [41], and filtered for SNPs (vt v0.5772-60f436c3) [55], a total read depth exceeding 100 over all 36 haplotypes, and a phred scaled quality score of at least 30. Because freebayes is capable of calling multi-nucleotide polymorphisms [54], we used a customized R-script (R v.3.5.3 [56]) to decompose the result in single-base resolution.

Four out of 36 haplotypes were excluded from the main analysis: 2 haplotypes displayed only homozygous genotype calls, suggesting that these two particular crosses were not successful (e.g., the supposedly virgin female was already mated); 2 haplotypes, which were generated by a recombination event between class 1 and class 2, were excluded from the analysis to obtain marker SNPs at the *Ace* locus that allow to unambiguously distinguish haplotype class 1 from haplotype class 2 (Additional file 1: Figure S3). Based on the remaining 32 haplotypes, we detected 166 marker SNPs between haplotype class 1 and class 2. One hundred twenty-six out of 166 marker SNPs were part of the Pool-seq SNP set of the experimental *D. simulans* population in the hot regime and 128 out of 166 marker SNPs in the cold regime. Forty (hot regime)/38 (cold regime) marker SNPs were excluded from the analysis, because they had insufficient coverage in at least one Pool-Seq sample. We used the median frequency of the remaining 126 (hot regime)/128 (cold regime) marker SNPs to assess the frequencies of the haplotype classes.

### Haplotype class analysis

We found pronounced haplotype structure around the *Ace* locus in the founder population and determined three distinct haplotype classes. Haplotype class 1 consists of two sub-groups (1a and 1b) with few segregating variants, while haplotype class 2 (*n* = 5) has normal levels of variation [14]. Haplotype class 1a (*n* = 19) differs from haplotype class 1b (*n* = 8) by carrying the three resistant mutations I161V, G265A, and F330Y. The nucleotide diversity $\pi =\left(\frac{1}{\frac{n\times \left(n-1\right)}{2}}{\sum}_{i=1}^n{\sum}_{j>i}^n{d}_{ij}\right)\times \frac{1}{L}$ within haplotype classes was calculated with R. *n* represents the total number of DNA sequences, *d*_*ij*_ is the number of nucleotides that differ between sequences *i* and *j*, and *L* is the total number of nucleotides examined (*L* = 36265, length of the *Ace* gene).

We used the median frequency of 126 (hot regime)/128 (cold regime) marker SNPs that distinguish haplotype classes 1 and 2 in combination with I161V, G265A, and F330Y to assess the frequencies of the three haplotype classes in the experimental *D. simulans* populations.

Replicate-specific selection coefficients for the haplotype classes were calculated with the R-package poolSeq (v 0.3.2) [49], assuming co-dominance and using the replicate-specific effective population size estimates (*N*_*e*_ hot regime = 252, 229, 268, 209, and 245; *N*_*e*_ cold regime = 209, 255, 259, 198, and 175*)* [35]. The measured selection coefficient of haplotype class 1a is the combined effect of the three resistance mutations and the selection coefficient of the haplotype class on which they are located (1b). Thus, we estimated the selection coefficient of the three resistance mutations by subtracting the *s* estimate of 1b from 1a.

### Insecticide bioassays

We tested the resistance of reconstituted ancestral [57] and evolved (generation 132) *D. simulans* populations to two different insecticides both targeting AChE: the carbamate propoxur (10 μg) and the organophosphate malathion (2 μg) (Pestanal Sigma-Aldrich). The bioassays were conducted in 30-ml glass vials coated with 250-μl acetone-insecticide dilution. To assure a uniform distribution of the insecticide, the vials were swirled until complete evaporation of the acetone-insecticide dilution and were left under a fume hood for 1 h before the assay. For a single measurement, we put 30 3–5-day-old females into a vial sealed with a cotton ball that was moistened with 1.5 ml of 5% sucrose solution and recorded mortality after 24 h insecticide exposure (23 °C, 70 to 80% relative humidity). We compared four replicates of the reconstituted ancestral population with three replicates of each evolved replicate. To measure insecticide resistance levels, we first determined the susceptibility for the sensitive *Ace* alleles from the *D. simulans* M252 reference strain [15] and resistant alleles from the reconstituted ancestral population. We estimated the dose resulting in 50% mortality after 24 h (LD_50_) for M252 and the ancestral population using five different concentrations and four replicates for each insecticide and population. The mortality of the reference strain M252 was assessed for 0.1, 0.3, 0.5, 0.7, and 1 μg propoxur and 0.01, 0.05, 0.1, 0.5, and 1 μg malathion. The ancestral population was exposed to 1, 2, 10, 20, 60, and 90 μg propoxur and 2, 5, 10, 20, and 30 μg malathion. Probit analysis implemented in the drc R package (version 3.0-1 [58]) were conducted to estimate LD_50_ in both populations.

The insecticide doses for the main bioassay (10 μg propoxur, 2 μg malathion) were chosen such that they did not exceed the LD_50_ of the ancestral population (LD_50_ propoxur = 19.5 μg, LD_50_ malathion = 3.4 μg).

### Phenotypic assays

We measured two fitness proxies for 11 lines being homozygous at the *Ace* locus: fecundity and egg-to-adult viability. Each of the 11 lines was made homozygous for the *Ace* locus by using the descendants of a single brother-sister cross, for which the identity of the haplotype class and the homozygosity was confirmed by Sanger sequencing. All phenotypic assays were conducted in a common garden setting to avoid uncontrolled environmental variation confounding the measurements. Prior to the phenotypic assays, the eleven assessed lines (six lines of haplotype class 1a and five lines of haplotype class 1b) were amplified at 20 °C with a 12-h dark/light cycle and maintained for one generation under density-controlled conditions (400 eggs per bottle) to avoid maternal effects of different densities. The statistical analysis of the two assessed phenotypes was conducted in R (version 3.5.3 [56]). To avoid biased treatment of lines based on the anticipated outcome of the phenotypic assays, researchers involved in the maintenance and the phenotyping of the lines were blinded for the haplotype class of each line.

We measured fecundity and viability in two different temperature regimes: a hot regime (12-h light and 28 °C; 12-h dark and 18 °C) and a cold regime (12-h light and 20 °C; 12-h dark and 10 °C).

#### Fecundity

Fecundity was measured for two replicates per line and temperature, resulting in 44 samples. After one generation of density control, 100 3-day-old flies were put into embryo collection cages (petri dishes with a diameter of 100 mm) on high-contrast media [59]. We assessed the number of laid eggs per embryo collection cage over the course of 11.5 days with an automatized approach [59]. After the fecundity assay, females were counted and fecundity was defined as the average number of eggs per female. One line (belonging to haplotype class 1a, 4 samples) was excluded from the analysis, because more than half of the flies were lost in one of the line-specific replicates during transfers.

To assess the impact of haplotype class and temperature, we fitted a linear mixed model [60] using function lmer() in the R package lme4 (version 1.1-21 [60]) with log_10_-transformed average number of eggs per female as a response. Haplotype class, temperature, both dummy coded, and their interaction were included as fixed effects into the model. A random intercept was fitted for each line to model the covariance structure in our data. The interaction of temperature and haplotype class is considered the focal term in the model. All assumptions for linear (mixed) models were met—residuals and random intercept effects were normally distributed and residuals displayed variance homogeneity. The absence of collinearity was confirmed via generalized variance inflation factors [61] calculated using the vif() function in the R package car (version 3.0–5 [62]).

Model stability was confirmed with a leave-one-out cross-validation of random effect levels with a custom R script. Confidence intervals were determined with parametric bootstrapping (*n* = 1000) with a custom R script. We tested the significance of fixed effects and their interaction with likelihood ratio tests [63] using the drop1() function with the parameter test set to “Chisq.”

#### Egg-to-adult viability

Egg-to-adult viability was measured in 14 replicates per line and temperature regime (generated in 7 batches over the course of 11 days), except for one line in haplotype class 1b in the hot regime with only 12 replicates, resulting in 306 samples. After one generation of density control, 250 2–4-day-old flies were put into small embryo collection cages (petri dishes with a diameter of 60 mm containing egg-laying medium: 4% agar and 4% sucrose with 1 ml yeast paste) [59] and were maintained at 20 °C in a 12-h dark/light cycle. Petri dishes were changed twice per day. Eggs were collected after 14 h of oviposition, and 60 eggs were transferred to vials containing the same Drosophila medium that was used in the E&R study. Vials were checked daily for their developmental state. Once flies started to eclose (day 10 after setup in the hot regime, day 22 after setup in the cold regime), freshly eclosed flies were collected for each replicate twice per day for seven consecutive days in the hot regime and for eight consecutive days in the cold regime, frozen at − 20 °C, and counted afterwards. Viability was defined as the total number of eclosed flies per vial over the whole assaying period.

To assess the influence of temperature, haplotype class, and their interaction on viability, we fitted a generalized linear mixed model with binomial error structure and logit link function [64] using the glmer() function in the R package lme4 (version 1.1-21 [60]). Egg to adult viability was treated as binomial proportions coded as a matrix with two columns, containing the number of eclosed flies (successes) in the first column and the number of not developed eggs (= 60 − number of eclosed flies) (failures) in the second column. Haplotype class, temperature (both dummy coded), and their interaction were fitted as fixed effects. A model with a combined line and batch random intercept effect was slightly overdispersed (*λ* = 1.455). We calculated the dispersion factor *λ* with a custom R script. To ensure the absence of overdispersion, we fitted an observation level random effect (*λ* = 0.301). Given that this model is under-dispersed, the resulting *p* values are conservative. The interaction between haplotype class and temperature is considered the key component of the model.

Significance of the interaction between haplotype class and temperature was assessed via a likelihood ratio test comparing the full model including the interaction with a reduced model that contains all components and the observation level random effects of the full model, except the interaction of haplotype class and temperature. We confirmed model stability, absence of collinearity, and calculated confidence intervals for the fixed effects as described for the fecundity phenotype. Best linear unbiased predictions for random effects were normally distributed. Linear predictors (LP) were transformed to probabilities via the inverse logit transformation: ${p}_{\mathrm{success}}=\frac{e^{\mathrm{LP}}}{1+{e}^{\mathrm{LP}}}$.

## Supplementary information

**Additional file 1: Table S1.** Influence of insecticide resistance mutations on fecundity. **Table S2.** Influence of insecticide resistance mutations on viability. **Figure S1.** Marker SNPs trajectories in the hot regime. **Figure S2.** Marker SNPs trajectories in the cold regime. **Figure S3.** Haplotype structure at the *Ace* locus.

## Data Availability

Information regarding data availability and processing steps of the experimental *D. simulans* populations can be found in Mallard et al. [34] and Otte et al. [35]. Raw reads for the ancestral haplotypes are available from the European Nucleotide Archive under project accession number PRJEB39894 [65]. Allele frequencies of the experimental *D. simulans* populations around the *Ace* locus (± 200 kb), CMH test results around the *Ace* locus (± 200 kb), estimated effective population sizes, SNP data sets and genotypes (vcf format) for the ancestral haplotypes, frequencies of haplotype classes, phenotypic data, and all scripts are available from the Dryad Digital Repository (10.5061/dryad.w0vt4b8p2) [66].
