# Supplementary material for: Fitness effects for Ace insecticide resistance mutations are determined by ambient temperature
Source: BMC Biol. 2020 Oct 30;18:157. doi: 10.1186/s12915-020-00882-5 (PMC7597021; doi:10.1186/s12915-020-00882-5)
Supplement: Supplementary file 1 — Additional file 1: Table S1. Influence of insecticide resistance mutations on fecundity. Table S2. Influence of insecticide resistance mutations on viability. Figure S1. Marker SNPs trajectories in the hot regime. Figure S2. Marker SNPs trajectories in the cold regime. Figure S3. Haplotype structure at the Ace locus. [file 12915_2020_882_MOESM1_ESM.pdf]

Supplementary Tables

Table S1 Influence of insecticide resistance mutations on fecundity

term	est.	SE	min	max	χ^2	df	P
intercept	1.295	0.029	1.280	1.239			[3]
class[1]	0.115	0.041	0.103	1.354			[3]
temperature[2]	0.203	0.032	0.186	1.443			[3]
class***temperature**	-0.007	0.046	-0.031	0.015	0.025	1	0.8752

*Results of the Linear Mixed Model with log_{10} transformed fecundity as response variable. est. = estimated effect size; SE = standard error; min/max = range of estimates obtained with leave one out cross validation of random effects; χ^2 = χ^2 value of a likelihood ratio test comparing full (with class*temperature interaction) and null model (without class*temperature interaction); df = degrees of freedom for the full-null model comparison; P = p-value for the full-null model comparison.*

[1] dummy coded with haplotype class 1b as reference category

[2] dummy coded with the cold regime as reference category

[3] not shown because of having a very limited interpretation

Table S2 Influence of insecticide resistance mutations on viability

term	est.	SE	min	max	χ^2	df	P
intercept	0.546	0.060	0.526	0.561			[3]
class[1]	-0.763	0.081	-0.778	-0.744			[3]
temperature[2]	-0.872	0.086	-0.896	-0.852			[3]
class***temperature**	0.444	0.116	0.424	0.469	14.479	1	<0.001

*Results of the Generalized Linear Mixed Model with viability as response variable. est. = estimated effect size (logit transformed); SE = standard error; min/max = range of estimates obtained with leave one out cross validation of random effects; χ^2 = χ^2 value of a likelihood ratio test comparing full (with class*temperature interaction) and null model (without class*temperature interaction); df = degrees of freedom for the full-null model comparison; P = p-value for the full-null model comparison.*

[1] dummy coded with haplotype class 1b as reference category

[2] dummy coded with the cold regime as reference category

[3] not shown because of having a very limited interpretation

Supplementary Figures

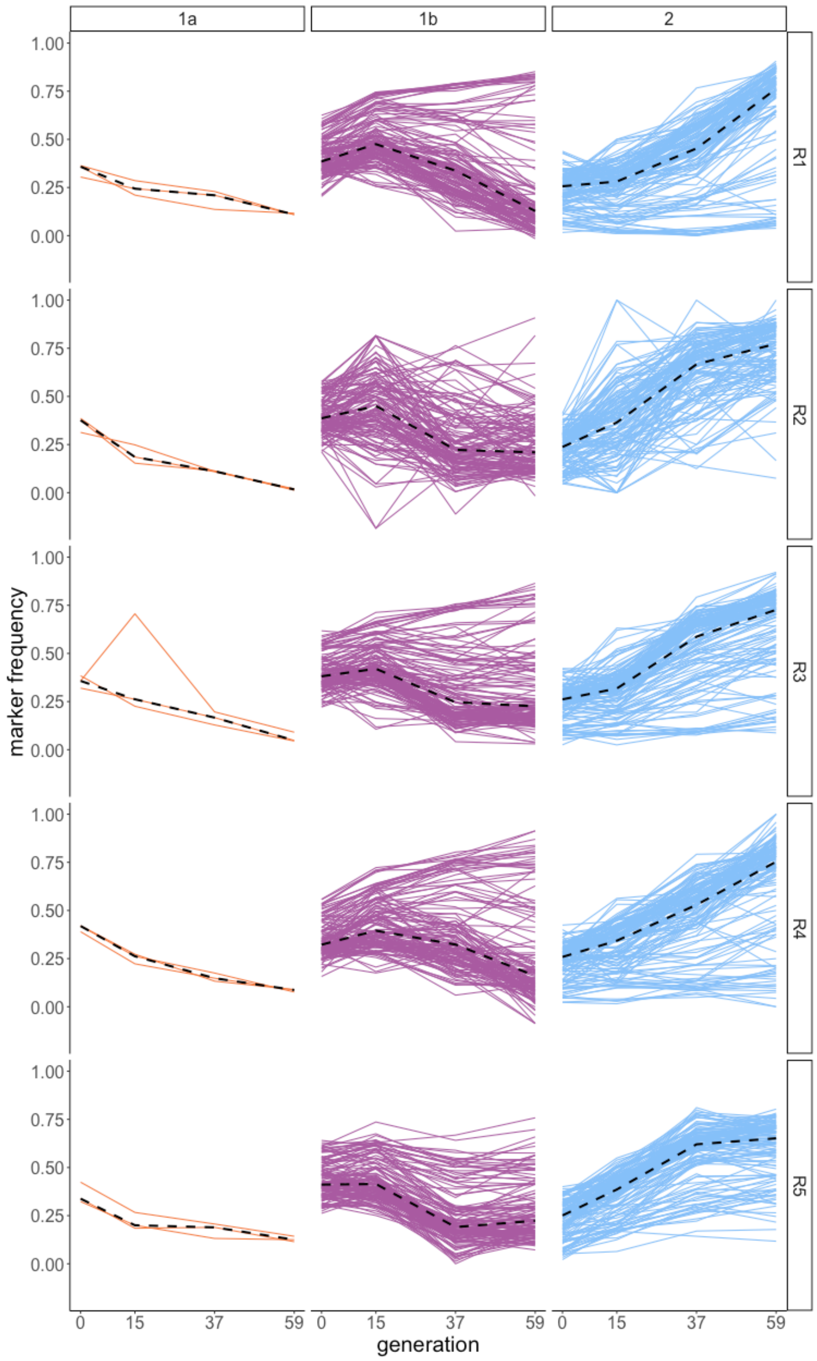

Figure S1 Marker SNPs trajectories in the hot regime. In each experimental population, the haplotype class frequency (= black dashed line) is determined by the median frequency of all marker SNPs (= colored lines). Each row shows one experimental population replicate.

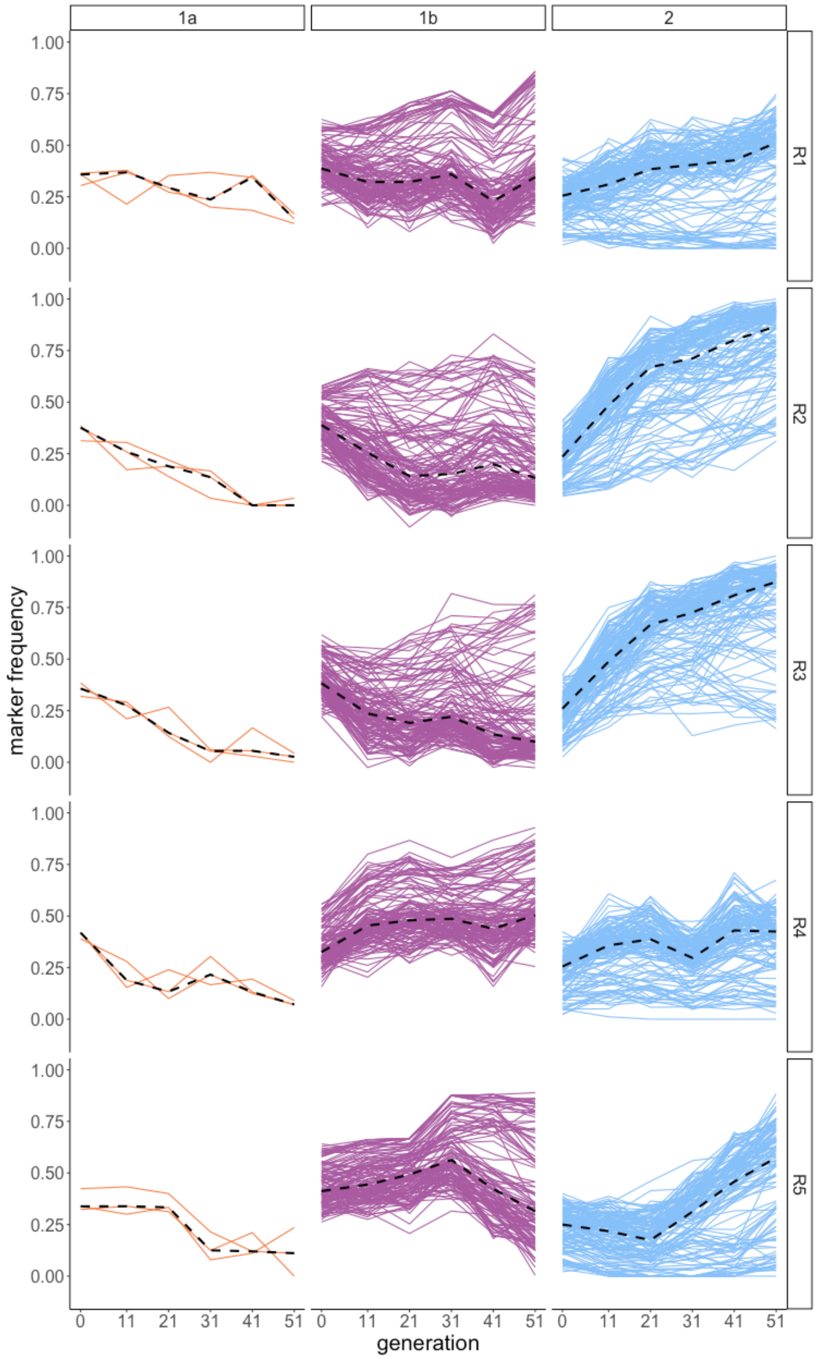

Figure S2 Marker SNPs trajectories in the cold regime. In each experimental population, the haplotype class frequency (= black dashed line) is determined by the median frequency of all marker SNPs (= colored lines). Each row shows one experimental population replicate.

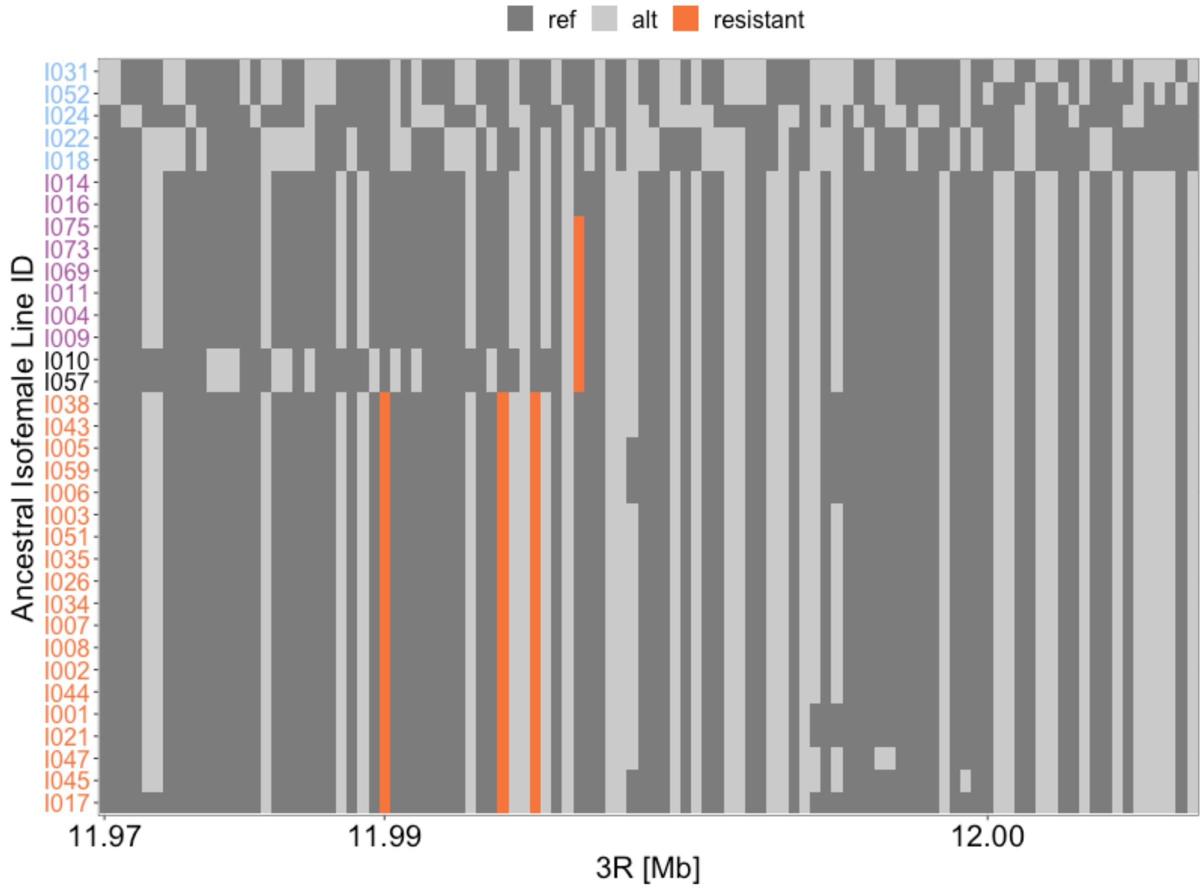

Figure S3 Haplotype structure at the Ace *locus: The x-axis shows the genomic position on chromosome 3R in mega-bases (Mb) around the* Ace *locus. Each single row shows the haplotype information of one isofemale line originating from the natural Portuguese* D. simulans *population. Isofemale line identifiers are colored by the assigned haplotype class (2 in blue, 1a in orange, and 1b in magenta), where recombinant lines are shown in black. Each column represents one exonic SNP at the* Ace *locus. Reference alleles (M252) are colored in dark grey, alternative alleles in light grey. The four previously reported resistant mutations I161V, G265A, F330Y, and G368A are highlighted in orange, with the three mutations I161V, G265A, and F330Y being specific to haplotype class 1a. Haplotypes I010 and I057 were excluded to assess marker SNPs that unambiguously distinguish class 1 from class 2.*
